# Citric Acid Cycle Genes and Nutrigenetics

**DOI:** 10.3390/ijms27052360

**Published:** 2026-03-03

**Authors:** Anna Vesnina, Oksana Kozlova, Svetlana Ivanova, Alexander Prosekov

**Affiliations:** 1 DNA Sequencing and Genomics Laboratory, Kemerovo State University, Krasnaya Street, 6, Kemerovo 650043, Russia; 2 Food Technology Institute, Kemerovo State University, Kemerovo 650043, Russia; ms.okvk@mail.ru; 3 Institute of NBICS-Technologies, Kemerovo State University, Krasnaya Street, 6, Kemerovo 650043, Russia; pavvm2000@mail.ru; 4 Department of TNSMD Theory and Methods, Kemerovo State University, Krasnaya Street, 6, Kemerovo 650043, Russia; 5 Laboratory of Biocatalysis, Kemerovo State University, Krasnaya Street, 6, Kemerovo 650043, Russia; a.prosekov@inbox.ru

**Keywords:** citric acid cycle, TCA cycle, Krebs cycle, mitochondria, epigenetics, metabolism, nutrition, diet

## Abstract

The citric acid cycle disruptions are implicated in the pathogenesis of chronic diseases, including diabetes, obesity, cancer, and cardiovascular conditions. Numerous publications link TCA cycle disorders to oncological, neurodegenerative, and osteoporotic diseases, and specific single-nucleotide polymorphisms have been proposed as potential markers. Nevertheless, lifestyle and diet have been strongly linked to risk factors for mitochondrial dysfunction; thus, preventive measures that minimize these risks are a relevant field of research. This review summarizes 45 years of relevant publications on the TCA cycle, its genetics and epigenetics, and the restorative potential of certain nutrients. The review includes articles in English and Russian, registered in PubMed, Elsevier, eLIBRARY.RU. The genes encoding the TCA cycle enzymes have been collected and presented. Information is provided that a number of changes in the expression of these genes, for example, Arg18Trp, Ser87Leu, Ala252Thr, and Leu357Val of the *ACO2* gene, leads to the development of neurodegenerative diseases; mutations rs121913499, rs121913500 in the *IDH1*, *IDH2* genes, rs1270341616 and the *DLST* gene lead to the development of cancer. There is evidence that through epigenetic modifications, nutrition affects the activity of the TCA cycle. Niacin, α-lipoic acid, succinic acid, resveratrol, curcumin, arginine, leucine, quercetin, ursolic acid, and alternol affect the regulation of the TCA cycle at the genetic level. Further research into the effects of plant metabolites, vitamins, and bioactive supplements on the TCA cycle may improve the existing preventative and therapeutic diets.

## 1. Introduction

**Figure 1:**
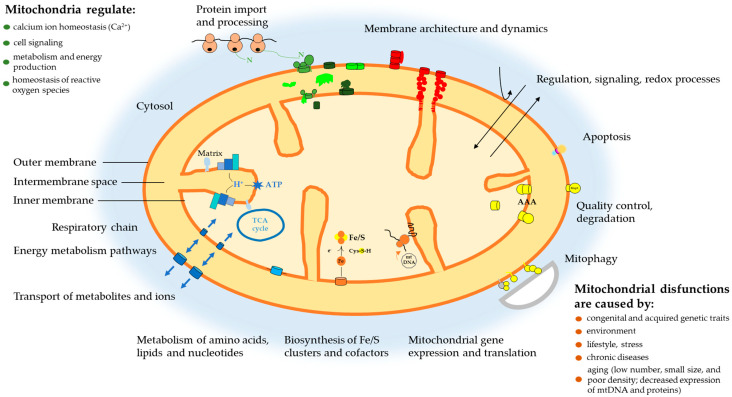
Mitochondria: functions and disorders [5,7,8].

Mitochondria are crucial for cellular processes. They host two mechanisms that underlie the metabolic pathways of most living organisms. Oxidative phosphorylation (OXPHOS) takes place in mitochondria. The citric acid cycle, also known as the tricarboxylic acid (TCA) cycle or Krebs cycle, occurs in the mitochondrial matrix. Discovered in 1937 by H. A. Krebs and W. A. Johnson [1,2,3,4], the TCA cycle is a fundamental respiration stage for all living cells: it provides the body with energy and maintains metabolism (Figure 1) [1,5,6].

Disturbances in the TCA cycle cause mitochondrial dysfunction that may trigger obesity, diabetes, cardiovascular diseases, cancer, and premature aging. Moreover, it may be closely related to neurodegenerative conditions such as Alzheimer’s and Parkinson’s diseases [9,10,11,12].

A proper diet may prevent mitochondrial dysfunction and, consequently, a number of chronic diseases [13,14]. The ketogenic and Mediterranean diets positively impact mitochondrial function [15,16,17]. The Western diet, on the contrary, contributes to mitochondrial dysfunction due to plant fats, sugars, and monosodium glutamate [18].

This article is a review of the scientific publications on various issues related to the TCA cycle, e.g., its intermediates that regulate the epigenetic landscape of gene expression, nutrients and diets that affect the TCA cycle and cellular metabolism.

Generalization of the available data can contribute to a deeper understanding of the potential directions of dietary and metabolic correction that prevent violations of the tricarboxylic acid cycle. However, the practical implementation of preventive strategies requires further interdisciplinary research and accumulation of clinical evidence.

## 2. TCA Cycle: Concept and Role in Healthy Metabolism

**Figure 2:**
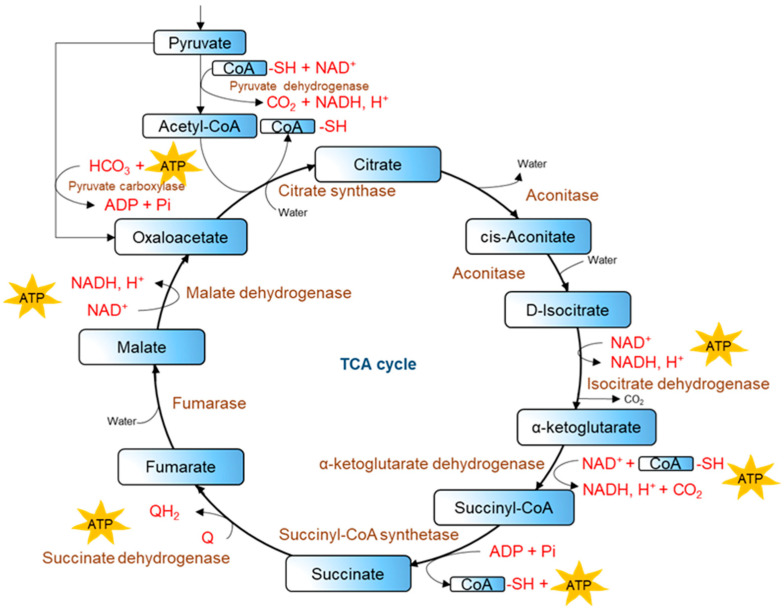
Simplified TCA cycle scheme.

The TCA cycle is a core part of cellular respiration: it is a cyclical pathway of enzymatic reactions inside mitochondria (Figure 2). As organic molecules of carbohydrates, proteins, and fats oxidize, they release energy in the form of adenosine triphosphate (ATP) [19].

As a TCA cycle component, pyruvate (pyruvic acid) appears during glycolysis. Glycolysis is a central metabolic pathway used by all cells during glucose oxidation to produce energy in the form of adenosine triphosphate and intermediates to serve other metabolic pathways [2].

Arnold et al. [2] described the processes of cataplerosis and anaplerosis. Cataplerosis is responsible for removing TCA cycle intermediates to support other metabolic pathways. Anaplerosis replaces these intermediates to maintain continuous oxaloacetate formation (Table 1).

Anaerobic glycolysis occurs without oxygen. Aerobic glycolysis involves oxidative decarboxylation of pyruvate to water and carbon dioxide in the presence of oxygen; i.e., decarboxylated pyruvate combines with coenzyme A and enters the TCA cycle [20,21].

The TCA cycle consists of eight stages, each with its own intermediate, namely citrate, isocitrate, oxoglutarate, succinyl-CoA, succinate, fumarate, malate, and oxaloacetate. The final products include carbon dioxide (CO_2_), nicotinamide adenine dinucleotide (NADH), flavin adenine dinucleotide (FADH_2_), and adenosine triphosphate (ATP) [19,22].

The TCA cycle reactions are carried out by such enzymes as citrate synthase (CS), aconitase (ACO2), α-ketoglutarate dehydrogenase (OGDH), succinate dehydrogenase (SDH), malate dehydrogenase (MDH), fumarase (FH), isocitrate dehydrogenase (IDH), and succinyl-CoA synthetase. Almost all enzymes of the tricarboxylic acid cycle are localized in the mitochondrial matrix. The exception is succinate dehydrogenase (SDH), which is associated with the inner mitochondrial membrane and simultaneously functions as part of the respiratory chain [19,23].

As part of its functions, the TCA cycle releases energy as adenosine triphosphate (ATP), produces electron carriers NADH and FADH_2_, participates in redox homeostasis, and synthesizes intermediates for metabolic pathways. Disturbances in the TCA cycle can alter cellular energy homeostasis and redox balance, potentially contributing to mitochondrial dysfunction and oxidative stress. Such metabolic alterations have been reported in association with neurodegenerative disorders, including Alzheimer’s disease [24]; however, current evidence mainly points to a correlation rather than a direct causal relationship. Furthermore, impaired TCA cycle progression can lead to the accumulation of intermediate metabolites such as 2-hydroxyglutarate (2-HG), succinate, and fumarate, which are known to exert epigenetic and signaling effects and are therefore termed oncometabolites [2,25,26,27]. Metabolic reprogramming involving TCA-related pathways has also been observed in osteoporosis, cardiovascular disease [28,29,30], and thyroid disease [3], although the underlying mechanisms remain poorly understood and are likely multifactorial.

De Castro Fonseca et al. [28] reported that succinate acts as an extracellular ligand that binds to GPR91, a G protein-coupled receptor associated with kidneys, liver, heart, retinal cells, and possibly many other tissues. The process may result in a wide range of physiological and pathological effects, which means that the TCA cycle is a crucial factor in healthy metabolism.

A disrupted TCA cycle and the resulting mitochondrial dysfunction are associated with poor nutrition. Excessive calorie consumption disrupts the balance of adenosine triphosphate, leading to oxidative stress, inflammation, and impaired cell apoptosis. Malnutrition or a diet low in vitamins B and minerals may disrupt the function of enzymes, leading to obesity, diabetes, cardiovascular diseases, joint and bone health issues, and autoimmune conditions [22].

A number of genetic traits pose a risk for the TCA cycle; a tailored diet may prevent mitochondrial dysfunction, which makes nutrigenomics a highly relevant field of medical research [7].

## 3. Genes That Encode TCA Cycle Enzymes

**Figure 3:**
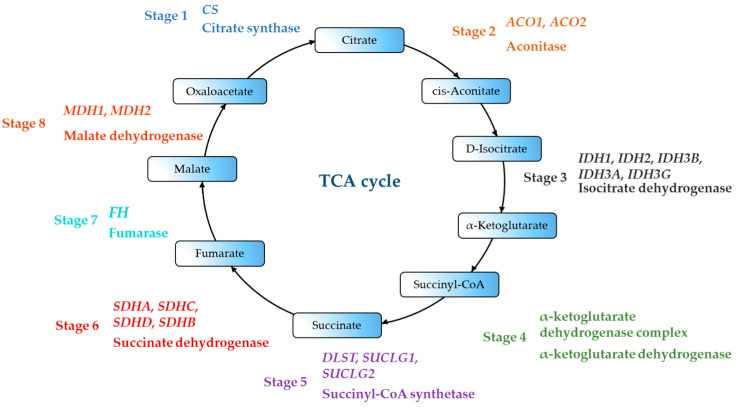
TCA cycle genes. The figure shows the 8 stages of the TCA cycle, the key enzymes and the genes that encode them.

Figure 3 shows the genes encoding the enzymes of the TCA cycle.

The genes shown in Figure 3 are shown in Table 2. Table 2 shows the genes directly or indirectly involved in the TCA cycle.

Scientific evidence has been found that certain changes in the expression of TCA cycle genes influence the development of neurodegenerative diseases and oncology. In the work of J. Zhu [65], clinical studies have shown that variants Arg18Trp, Ser87Leu, Ala252Thr and Leu357Val of the *ACO2* gene can be used as a biomarker in Parkinson’s disease. The work of R. Spiegel [66,67] has shown that the Ser112Arg mutation in the *ACO2* gene leads to a neurodegenerative disease called cerebellar atrophy. Mitochondrial dysfunction is strongly associated with cancer [68]. Nölting et al. [69] linked TCA cycle gene mutations to pheochromocytoma or paraganglioma. Cho et al. [70] explained individual differences in energy balance by genetic polymorphisms in mitochondria, which affect energy metabolism at the cellular level. The team identified single nucleotide polymorphisms (SNPs) of the TCA cycle associated with colorectal cancer risk in the UK. Mutations in the *IDH1* and *IDH2* genes (rs121913499, rs121913500) lead to the formation of an oncometabolite, 2-hydroxyglutarate. This metabolite causes hypermethylation of histones and DNA, leading to tumor cell formation in vitro [71,72]. This oncometabolite also accumulated with the mutation c.1121G > A (p.Gly374Glu, rs1270341616) of the DLST gene [59]. Mutations in the *FH* gene lead to high levels of intracellular fumarate, which contributes to cancer development. Mutations in the TCA gene cause the accumulation of oncometabolites, triggering carcinogenic processes. Understanding these genetic characteristics can aid in studying the mechanisms of cancer development and in developing diagnostic methods [73].

Epigenetic modifications play a special role in understanding the development of various diseases [74].

### 3.1. Epigenetics of the TCA Cycle

Epigenetic modifications alter gene expression without changing their sequence [75]. They are dynamic, flexible, and reversible under external stimuli [76]. Epigenetic mechanisms include nucleosome remodeling and regulatory non-coding (small and micro) RNAs. However, the main key epigenetic modifications are DNA methylation and reversible histone modifications, e.g., acetylation, methylation, and ubiquitination [77].

DNA and histone methylation are the key epigenetic tools that regulate gene expression. DNA methylation is a covalent modification of DNA: a methyl group (-CH3) binds with cytosine groups in DNA as part of a CpG dinucleotide at C5 of the cytosine ring. DNA methylation is carried out by DNA methyltransferase (DNMT) enzymes, such as DNMT1, DNMT3A, and DNMT3B [78]. They catalyze the transfer of a methyl group from S-adenosyl-L-methionine (SAM), the ubiquitous methyl group donor, to the fifth position of the cytosine pyrimidine ring [79,80,81,82].

2-Ketoglutarate-dependent dioxygenase (2-OGDO) enzymes are DNA and histone demethylation enzymes, i.e., they remove methyl groups from DNA and histones. Such TCA cycle intermediates such as 2-oxoglutarate (ketoglutarate) are substrates for 2-OGDO enzymes. A 2-OGDO complex consists of three enzymes [81,83]:

- 2-oxoglutarate decarboxylase/2-oxoglutarate decarboxylase (E1);
- dihydrolipoamide succinyl transferase (E2);
- dihydrolipoamide dehydrogenase (E3).

The TCA cycle activity depends on the expression of 2-oxoglutarate dehydrogenase (2-OGDH) enzymes. The 2-OGDH occupies a central position at the interface of energy and epigenetic metabolism, since it controls the rate of conversion of 2-oxoglutarate to succinyl-CoA and thereby determines the intracellular pool of 2-oxoglutarate available for 2-OGDO [83]. It should be noted that 2-OGDH is not the only regulator of the tricarboxylic acid cycle, but is one of its key rate-limiting enzyme units along with citrate synthase and isocitrate dehydrogenase.

Low succinate dehydrogenase (SDH) leads to succinate accumulation. Succinate and fumarate are strong inhibitors of 2-OGDO enzymes. As Table 3 shows, this inhibition enhances DNA and histone methylation regulated by DNA methyltransferases (TET1–3) and histone methyltransferases (JmjC KDM2–7) [81].

According to Arnold et al. [2], metabolites formed in the TCA cycle are responsible for the regulation of chromatin. For instance, histone acetyltransferases change the accessibility of chromatin by transferring acetyl groups from acetyl-CoA to histones. Histone deacetylases remove acetyl groups from histones and produce acetate. α-Ketoglutarate-dependent dioxygenases regulate the demethylation of histones and nucleic acids, e.g., in *FTO* [98] and *ALKBH5*.

Other inhibitors include reactive oxygen species, metals (Co^2+^, Ni^2+^), and Fe^2+^ chelators. The list of cofactors includes NAD^+^, acetyl CoA, Ca^2+^, lipoic acid, thiamine, and ferrous iron (Fe^2+^). They can regulate the functional yield of 2-OGDH [81,82,83]. Therefore, the balance of TCA cycle reactions may affect DNA and histone methylation.

The work of J. Małecki [99] has demonstrated, using in vitro and in vivo approaches, that lysine methylation occurs in the CS gene at the Lys-395 residue. Methylation reduced the activity of the gene and was inhibited by the substrate oxaloacetate and adenosine. The authors hypothesized that methylation may regulate CS function in response to changes in metabolite levels. However, additional research is needed to confirm this hypothesis.

In the work of Z. Mao [100], the authors found that the activity of MDH2 is associated with an anti-aging mechanism. In vitro and in vivo studies showed that increased MDH2 expression led to increased cellular senescence. Inhibition of MDH2 expression increased histone methylation, which in turn slowed down cellular aging.

Lifestyle factors, in particular nutrition, affect the functioning of cells and the body through epigenetics. Therefore, the search for nutrients capable of epigenetic modifications is relevant [74,101].

#### Nutrients That Regulate the TCA Cycle

Nutraceuticals are biologically active substances included in beneficial added dietary supplements (BADS) and functional foods as part of preventive or therapeutic diets [101]. Such nutrients as magnesium, riboflavin, nicotinamide, and biotin affect the expression of mitochondrial enzyme genes through systems of transcriptional coactivators and sensors of cell energy status [102].

**Figure 4:**
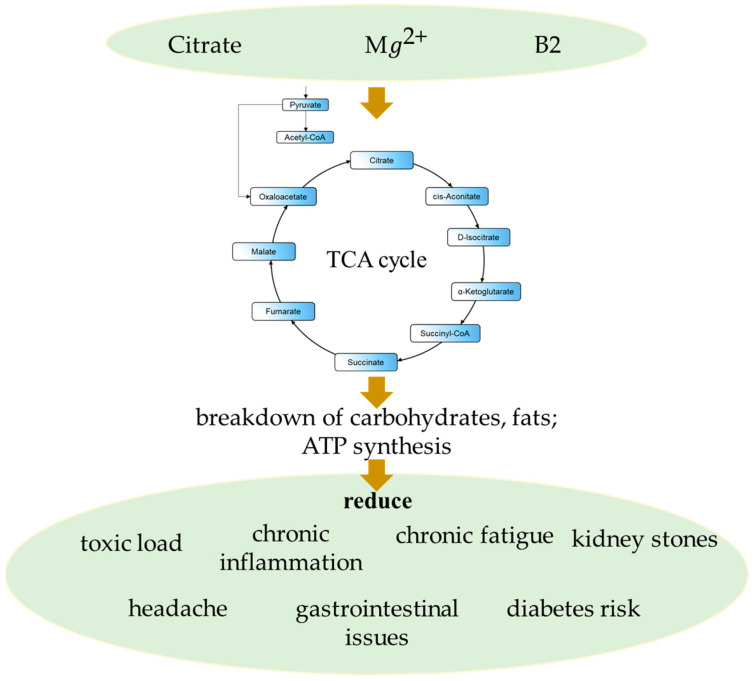
Synergistic effects of magnesium ion, citrate anion, and vitamin B2 [103].

**Figure 5:**
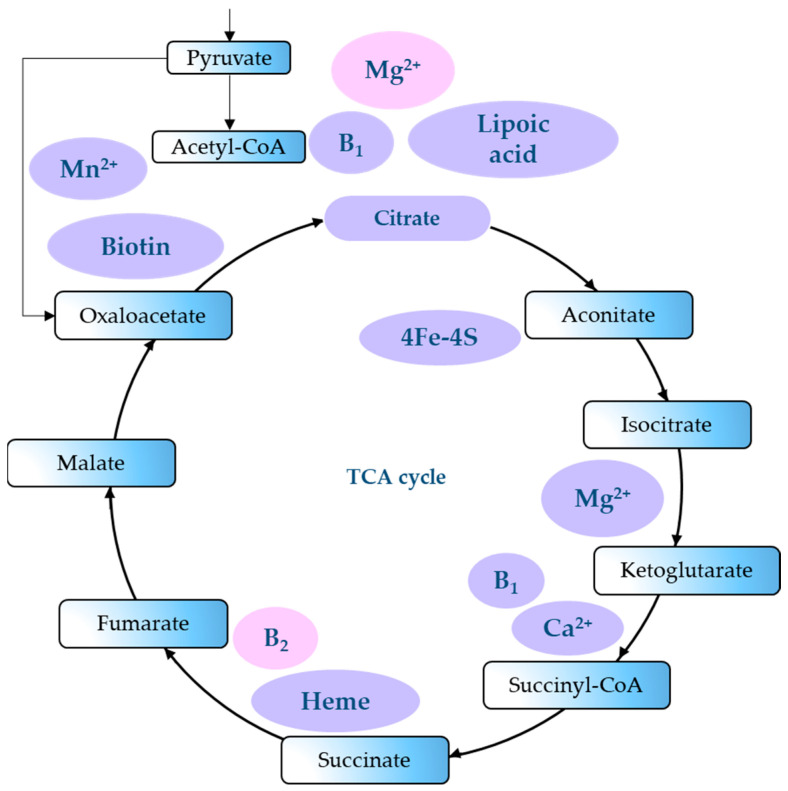
Effect of magnesium, citrate anion, and vitamin B2 on the TCA cycle [103].

Magnesium ion, citrate anion, and riboflavin are part of the TCA cycle (Figure 4 and Figure 5).

Magnesium is an essential cofactor for isocitrate dehydrogenase and pyruvate dehydrogenase phosphatase in the TCA cycle [103]. Riboflavin maintains the activity of the enzyme succinate dehydrogenase, which oxidizes succinate anion into fumarate anion [103]. Magnesium is a reliable and universal regulator since it provides the structural stability of the pyruvate dehydrogenase complex, isocitrate dehydrogenase, and α-ketoglutarate dehydrogenase. In addition, it affects the transcription of energy metabolism genes by activating AMP-activated protein kinase (AMPK), which is sensitive to intramitochondrial Mg^2+^ levels [103].

Other nutrients exercise a targeted effect on the mitochondrial transcriptome. As a precursor of NAD^+^, nicotinamide activates SIRT3/5 deacetylases, thus changing the expression and post-translational modifications of such enzymes such as SDHA, IDH2, and MDH2 [36].

Riboflavin (B_2_) forms flavin mononucleotide (FMN) and flavin adenine dinucleotide (FAD). It supports succinate dehydrogenase and acyl dehydrogenases. In addition, it controls the expression of mitochondrial genes under conditions of energy substrate deficiency [104].

Omega-3 polyunsaturated fatty acids stimulate the expression of PGC-1α, which enhances the mitochondrial biogenesis and increases the flow of acetyl-CoA through the TCA cycle [105]. In their study, A. I. Borja-Magno and colleagues [106] studied the effect of omega-3 fatty acids on mitochondrial bioenergetics. A case-control study was conducted involving 20 women with a normal body mass index and 19 women with grade 2 obesity, who took 5.25 g of omega-3 fatty acids for 1 month. The study found that taking omega-3 fatty acids improved mitochondrial dysfunction in peripheral mononuclear blood cells in obese individuals. There was a decrease in triglycerides, insulin, and IL-1b, IL-6 levels.

Resveratrol and curcumin activate SIRT1 and AMPK to increase oxidative metabolism [107].

Lipoic acid (ALA) is a cofactor for mitochondrial metabolism, specifically the enzymes PDH, OGDH, branched-chain ketoacid dehydrogenase, and 2-oxoadipate dehydrogenase play a key role in carbon entry into the Krebs cycle [108]. A similar role is played by coenzyme Q (CoQ, ubiquinone), which is a cofactor of mitochondrial dehydrogenases—dihydroorotate dehydrogenase, involved in the biosynthesis of pyrimidines, and flavoprotein dehydrogenase, involved in the beta-oxidation of fatty acids [109].

Therefore, dietary supplements containing ALA, CoQ, vitamin B2, magnesium, and omega-3 are important to support healthy body function.

Table 4 summarizes the nutrients that affect the expression of TCA cycle genes.

Nutrients and biologically active substances act as cofactors for TCA cycle enzymes while affecting its regulation at the genetic level, thus shaping the adaptive metabolic response.

Nutrients use a variety of molecular mechanisms to modulate TCA cycle genes, from direct coenzyme support to activating energy sensors and transcriptional coactivators. Magnesium, riboflavin, nicotinamide, and biotin primarily support the catalytic activity and stability of TCA cycle enzymes, attracting substrates and improving the oxidative metabolism. ALA, omega-3 PUFAs, resveratrol, curcumin, arginine, leucine, and quercetin activate the AMPK–SIRT–PGC-1α signaling pathway, which boosts mitochondrial biogenesis, improves energy output, and reduces oxidative stress. Nutrients are important for mitochondrial metabolism at the level of genetic transcription and post-translational modifications.

The exact impact of nutrients on TCA cycle genetics requires more research as they are connected with metabolic pathways and susceptibility to a number of socially significant diseases. New data make it possible to develop new preventive dietary recommendations, BADS, and functional foods [121].

Despite the bioactivity of nutrients and their metabolic significance, their application is rather limited. For example, a number of biologically active substances (resveratrol, curcumin, quercetin, ursolic acid) demonstrate low solubility or lipophilicity, which means poor bioavailability. Hydrophilic compounds cannot pass through the cell membrane; lipophilic compounds do not dissolve in the gastrointestinal tract; compounds are adsorbed only after being fermented by metabolites of gastrointestinal microbiota. In this regard, food science and medicine need more effective measures to increase the bioavailability of nutrients [101,122], e.g., by derivatization, exosomal and liposomal nanocarriers, and bioavailable derivatives [123].

## 4. Conclusions

Mitochondrial metabolism and the citric acid cycle provide cells with energy and maintain homeostasis. Mitochondrial dysfunction is a universal pathogenetic mechanism involved in a wide range of diseases, from neurodegenerative and metabolic to inflammatory [13]. Although all the biochemical stages of the TCA cycle were described back in 1937, we still have no clear understanding of how to control it. However, some nutrients (niacin, α-lipoic acid, succinic acid, resveratrol, curcumin, arginine, leucine, quercetin, ursolic acid, alternol) can modulate gene expression, thereby improving the efficiency of the TCA cycle, which makes this research direction extremely relevant.

This review left out publications on mitochondrial diseases caused by mtDNA defects and attempts to regulate the TCA cycle with hormones and signal transduction systems.

## 5. Strategies/Methods

This review covered 45 years of articles published in Russian and English and indexed in the PubMed (US National Library of Medicine), Scopus (Elsevier), and the Russian Scientific Electronic Library (eLIBRARY.RU). The final pool of the most relevant publications consisted of 123 articles.

The list of keywords included citric acid cycle, TCA cycle, Krebs cycle, mitochondrial dysfunction, TCA epigenetics, TCA genes, and mitochondrial health diet.

We focused on the papers describing the results of clinical and preclinical studies (regardless of the model objects), as well as review articles. We excluded the articles describing the results obtained in silico, conference proceedings, and monographs.

All the publications returned by the search were screened for relevance.

## Figures and Tables

**Table 1:** Cataplerosis and anaplerosis according to Arnold et al. [2].

Anaplerosis	
Product	Source
acetyl-CoA	Glucose oxidation
	Lactate oxidation
	Fatty acid oxidation
	Catabolism of amino acids, ketones, acetate
Cataplerosis	
Product	Source
oxaloacetate → asparate	Urea cycle
	Nucleotide synthesis
	Amino acid synthesis
oxaloacetate	Gluconeogenesis
citrate	Fatty acid synthesis
α-ketoglutarate	Glutamate → nucleotide synthesis
	Glutamate → amino acid synthesis
succinyl-CoA	Porphyrins

(→)—cause-and-effect relationship between processes.

**Table 2:** Genes directly or indirectly involved in the TCA cycle.

**No.**	**Gene**	**Enzyme**	**Function**	**Reference**
TCA Cycle				
Stage 1—Formation of citrate ion				
	*CS*	Citrate synthase (transferase)	Participates in the formation of citrate—catalyzes the synthesis of citrate from acetyl coenzyme A and oxaloacetate	[29]
Stage 2—Formation of isocitrate via cis-aconitate				
	*ACO1*	Aconitase 1 (lyase)	Its protein is a bifunctional “moonlighting” cytosolic protein that functions as an important enzyme in the tricarboxylic acid cycle and interacts with mRNA to control intracellular iron level.	[30]
	*ACO2*	Aconitase 2 (lyase)	Catalyzes the conversion of citrate to isocitrate via cis-aconitate	[31,32]
Stage 3—Oxidation of isocitrate to α-ketoglutarate				
	*IDH1*	Isocitrate dehydrogenase 1 (oxidoreductase)	Encodes isocitrate dehydrogenase 1, which converts isocitrate into 2-ketoglutarate; this reaction produces NADPH, which breaks down fats for energy and protects cells from reactive oxygen species	[33]
	*IDH2*	Isocitrate dehydrogenase 2 (oxidoreductase)	Catalyzes the oxidative decarboxylation of isocitrate to alpha-ketoglutarate with the formation of NADPH; crucial for intermediary metabolism and energy production; binds or interacts with the pyruvate dehydrogenase complex; undergoes alternative splicing into different transcript variants	[34,35,36]
	*IDH3B*	Isocitrate dehydrogenase [NAD] subunit beta (oxidoreductase)	Protein is the α-subunit and β-subunit of one isoenzyme of NAD(+)-dependent isocitrate dehydrogenase; the latter catalyzes the oxidative decarboxylation of isocitrate to α-ketoglutaric acid.	[37]
	*IDH3A*	Isocitrate dehydrogenase [NAD] subunit alpha (oxidoreductase)		[38]
	*IDH3G*	Isocitrate dehydrogenase [NAD] subunit gamma (oxidoreductase)	Plays an important role in the oxidative decarboxylation of isocitrate	[39,40]
Stage 4—Oxidation of α-ketoglutarate to succinyl-CoA				
	*OGDC (OGDHL)*	α-ketoglutarate dehydrogenase complex (KGDHc)	Catalyzes the oxidative decarboxylation of α-ketoglutarate to form succinyl-CoA and NADH; the reaction is virtually identical to the pyruvate dehydrogenase reaction of oxidative decarboxylation of pyruvate; pyruvate dehydrogenase (E1), dihydrolipoamide acetyltransferase (E2) and dihydrolipoamide dehydrogenase (E3).	[41,42,43,44,45,46,47]
	*DLD*	Dihydrolipoamide dehydrogenase (E3)	Encodes a self-assembling “moonlighting” protein that performs mechanistically distinct functions depending on its assembly state; in its homodimeric form, the protein functions as a dehydrogenase and is found in several multienzyme complexes that regulate energy metabolism; as a monomer, this protein can function as a protease.	[48]
Stage 5—Conversion of succinyl-CoA to succinate				
	*DLST*	Dihydrolipoyllysine-residue succinyltransferase component of 2-oxoglutarate dehydrogenase complex (transferase)	Catalyzes the conversion of succinyl-CoA to succinate	[49,50]
	*SUCL:*	Succinyl-CoA ligase (ligase)	Catalyzes the reversible conversion of succinyl-CoA and adenosine diphosphate (ADP) or guanosine diphosphate (GDP) to succinate and ATP or guanosine triphosphate (GTP), respectively	[51,52]
	*SUCLG1*	ATP-forming alpha subunit of succinate-CoA ligase (ligase)	Succinyl-CoA synthetase catalyzes a reversible reaction that produces succinyl-CoA and succinate	
	*SUCLG2*, *SUCLA2*	ATP-forming beta subunit of succinate-CoA ligase (ligase)		[33,51]
Stage 6—Oxidation of succinate to fumarate				
	*SDHA (SDH2)*	Succinate-ubiquinone oxidoreductase (oxidoreductase)	Encodes one of the four subunits in the succinate dehydrogenase enzyme complex, which is important for cellular respiration and the TCA cycle	[36,53]
	*SDHC*	Succinate dehydrogenase cytochrome b560 subunit (oxidoreductase)	Encodes one of the four nuclear-encoded subunits that comprise succinate dehydrogenase, i.e., mitochondrial complex II of the tricarboxylic acid cycle and the aerobic respiratory chains of mitochondria.	
	*SDHD*	Succinate dehydrogenase [ubiquinone] cytochrome b small subunit (oxidoreductase)	Responsible for the transfer of electrons from succinate to ubiquinone (coenzyme Q)	
	*SDHB*	Succinate dehydrogenase [ubiquinone] iron-sulfur subunit (oxidoreductase)		
Stage 7—Hydration of fumarate to malate				
	*FH*	Fumarate hydratase (lyase)	Catalyzes the reversible stereospecific conversion of fumarate to L-malate	[54]
Stage 8—Oxidation of malate to oxaloacetate				
	*MDH1*	Malate dehydrogenase 1 (oxidoreductase)	Encodes a crucial enzyme that catalyzes the NAD/NADH-dependent reversible oxidation of malate to oxaloacetate in a number of metabolic pathways, including the TCA cycle; catalyzes the reduction of aromatic alpha-keto acids in the presence of NADH	[55]
	*MDH2*	Malate dehydrogenase 2 (oxidoreductase)		[56]
Pyruvate dehydrogenase (PDH) complex				
	*DLAT*	Dihydrolipoamide acetyltransferase (E2 subunit) (transferase)	Encodes the E2 component of the multienzyme pyruvate dehydrogenase complex located in the inner mitochondrial membrane; there, it catalyzes the conversion of pyruvate to acetyl coenzyme A; its protein, dihydrolipoamide acetyltransferase, accepts acetyl groups formed by the oxidative decarboxylation of pyruvate and transfers them to coenzyme A.	[57]
	*DLST*	Dihydrolipoamide succinyltransferase (E2)		[58,59]
The PDH complex catalyzes the overall conversion of pyruvate to acetyl-CoA and CO_2_				
	*PDHA1*	Pyruvate dehydrogenase E1 component subunit alpha	A mitochondrial multienzyme complex that catalyzes the overall conversion of pyruvate to acetyl coenzyme A and carbon monoxide, linking glycolysis to the citric acid cycle	[60]
	*PDHA2*	Pyruvate dehydrogenase (NAD+)	Provides the activity of pyruvate dehydrogenase (NAD+) and pyruvate dehydrogenase (acetyl transfer enzyme); participates in pyruvate metabolism	[61]
	*PDHB*	E1-Β Subunit Of Pyruvate Dehydrogenase	Encodes E1-Β subunit; Its mutations are associated with pyruvate dehydrogenase E1-Β deficiency; has alternatively spliced transcript variants	[62]
	*PDHX*	E3-binding protein	Encodes the E3-binding protein subunit, also known as component X of the pyruvate dehydrogenase complex; physically links E3 dimers to the E2 core of the PDH complex	[63]
	*PDP1*	Catalytic subunit of pyruvate dehydrogenase phosphatase-1	Its kinases catalyze the phosphorylation of E1 serine residues to inactivate the E1 component and inhibit the complex; its phosphatases reverse the effects of kinases by catalyzing the dephosphorylation and activating the E1 component.	[64]

**Table 3:** Effect of 2-OGDH on epigenetic landscape [81].

**Enzyme**	**Target**	**References**
TET1, TET2, TET3	5mC hydroxylation (DNA)	[84,85]
KDM2 (JHDM1)	H3K36me1,2/H3K4me3 (histone H3)	[86,87]
KDM3 (JHDM2/JMJD1)	H3K9 me1,2 (histone H3)	[88,89]
KDM4 (JHDM3/JMJD2)	H3K9me2,3/H3K36me2,3 (histone H3)	[90,91]
KDM5 (JARID1)	H3K4me2,3 (histone H3)	[92,93]
KDM6 (JMJD3, UTX)	H3K27me2,3 (histone H3)	[94,95]
KDM7 (PHF2/8)	H3K9me1,2/H3K27me1,2 (histone H3)	[96,97]

**Table 4:** Nutrigenetics of the TCA cycle.

**Nutrient/BAS**	**Targeted/** **Modified Genes**	**Effect**	**Method of Action on the TCA Cycle**	**Biological Model**	**Reference**
Nicotinamide/niacin (B_3_), niacin derivative (acipimox)	*PDP*, *IDH3A*, *IDH3B*, *DLST*, *SUCLG2*, *CS*	↑ mitochondrial expression? ↑ NAD+ levels	↑ heat shock protein 60 (HSP60), the MTCO1 (mitochondrial-encoded cytochrome C oxidase I)/SDHA (succinate dehydrogenase complex, subunit A) ratio	C2C12 myoblasts, 3 h incubation with acipimox (10 mol/L)	[110]
			↓ cholesterol and triglyceride levels, ↑ nonesterified fatty acid levels, ↑ expression of metabolic genes (*PDP*, *IDH3A*, *IDH3B*, *DLST*, *SUCLG2*, *CS*)	21 patients with T2D, acipimox (250 mg three times daily) for 2 weeks	
α-lipoic acid (ALA)	*PDH*	regulation of mitochondrial redox balance	↑ PDH	CHO-DXB11 cell, ALA 10 _MK_M, 20 _MK_M, 50 _MK_M, 100 _MK_M, 200 _MK_M и 500 _MK_M, 120 h	[111]
Succinic acid/succinates	*HIF1A*, *IL1B*	↑ HIF-1α, transcription inhibition *IL1B*, *TNF-α*	stabilizes HIF-1α through succinate → alters the expression of metabolic genes	Female C57BL/6 mice	[112]
Resveratrol	*SIRT1*, *PGC-1α*,	↑ expression *SIRT1*, *PGC-1α*	↑ *SIRT1 →* *↑ PGC-1*α → ↑ TCA enzymes	male C57Bl/6J mice, 400 mpk dose	[107]
	*SIRT1*, *PGC-1α*, *NRF1*, *CYCS*	↑ mitochondrial mass, ↑ mitochondrial biogenesis	↑ *SIRT1*, *eNOS*	HCAECs cells, 1–10 μmol/L; for 24–48 h	[113]
Curcumin	*ERRα*, *HK1*, *HK2*, *PFKL*, *LDHA*, *ACO1*, *IDH2 u SUCLG2*	↓ ATP, ↓ expression ERRα, ACO1, IDH2 и SUCLG2, cholesterol content	↓ mitochondrial respiration (OXPHOS)	cells H295R, SW13, MUC-1, curcumin (0, 10, 20, 40, 80 µM) 24, 48, 72 h	[114]
Arginine supplement	*PGC-1α*, *SIRT1*	↑ SDH, ↑ mitochondrial biogenesis	↑ PGC-1α and SIRT1 → ↑ mtDNA	Duroc × Landrace × Yorkshire piglets, 0.5, 1.0 and 1.5% arginine, 4 weeks	[115]
	*PGC-1α*, *SDHA*	↑ expression PGC-1*α*, *SDHA*	ameliorated oxidative metabolism	17 male professional water polo, 5 g, 4 weeks	[116]
Leucine supplement	*SIRT1*, *AMPK*	↑ mitochondrial biogenesis	↑ SIRT1 and AMPK phosphorylation pathways	C2C12 myoblast cells, SH-SY5Y human neuroblastoma cell line	[117]
Quercetin	*PGC-1α*, *SIRT1*	↑ expression *PGC-1α*, *SIRT1*	activation of mitochondrial biogenesis	Male ICR mice, quercetin 12.5 or 25 mg/kg, 7 days	[118]
	*SIRT1*, *PGC-1α*, *TFAM*	↑ expression *SIRT1*, *PGC-1α*, *TFAM*		SH-SY5Y cells, quercetin (0, 2.5, 5, 7.5 и 10 μM), 24 h	[119]
Alternol	Enzymes: FH, MDH2, DLAT, DLST	↓ mitochondrial respiration and ATP production	Weakening of the enzymatic activity of TCA enzymes	cancer PC-3, 22RV1 cell lines, benign prostate BPH1 line, alternol, 10 μM for 4h	[120]

Note: (↑)—increases expression/activity; (↓)—reduces expression/activity; (→)—cause-and-effect relationship between processes; (↑ → ↑)—high activity of one regulator increases synthesis or activity of the other.

## Data Availability

No new data were created or analyzed in this study.

## Abbreviations

The following abbreviations are used in this manuscript:

TCA	the citric acid cycle, Krebs cycle, TCA cycle
OXPHOS	oxidative phosphorylation
ATP	adenosine triphosphate
NADH	nicotinamide adenine dinucleotide
FADH_2_	flavin adenine dinucleotide
CS	citrate synthase
ACO2	aconitase
OGDH	α-ketoglutarate dehydrogenase
SDH	succinate dehydrogenase
MDH	malate dehydrogenase
FH	fumarase
IDH	isocitrate dehydrogenase
SNP	single nucleotide polymorphisms
DNMT	DNA methyltransferase
2-OGDO	2-ketoglutarate-dependent dioxygenase
BADS	beneficial added dietary supplements
FMN	flavin mononucleotide
PDH	pyruvate dehydrogenase complex
KGDHc	α-ketoglutarate dehydrogenase complex
E1	pyruvate dehydrogenase
E2	dihydrolipoamide acetyltransferase
E3	and dihydrolipoamide dehydrogenase
ALA	α-lipoic acid
HSP60	heat shock protein 60
MTCO1	mitochondrial-encoded cytochrome C oxidase I
SDHA	succinate dehydrogenase complex, subunit A
